# Omega-3 fatty acids as host-directed immunomodulatory therapeutics in sepsis: real-world evidence supporting drug development potential for systemic inflammatory diseases

**DOI:** 10.3389/fcimb.2025.1738204

**Published:** 2026-01-26

**Authors:** Chengying Hong, Jinquan Xia, Zhenmi Liu, Yuting Chen, Kangping Hui, Wei Wang, Huaisheng Chen

**Affiliations:** 1 Department of Critical Care Medicine, Shenzhen People’s Hospital, Second Clinical Medical College of Jinan University, First Affiliated Hospital of Southern University of Science and Technology, Shenzhen, Guangdong, China; 2 Department of Clinical Medical Research Center, Shenzhen People’s Hospital, The Second Clinical Medical College, Jinan University The First Affiliated Hospital of Southern University of Science and Technology, Shenzhen, Guangdong, China; 3 Department of Critical Care Medicine, Shenzhen People’s Hospital, The Second Clinical Medical College, Jinan University, Shenzhen, China; 4 Endocrinology Department, Shenzhen People’s Hospital, Second Clinical Medical College of Jinan University, First Affiliated Hospital of Southern University of Science and Technology, Shenzhen, Guangdong, China; 5 Guangdong Provincial Clinical Research Center for Geriatrics, Shenzhen Clinical Research Center for Geriatrics, Department of Geriatrics, Shenzhen People’s Hospital, The First Affiliated Hospital, Southern University of Science and Technology, The Second Clinical Medical College, Jinan University, Shenzhen, Guangdong, China

**Keywords:** drug development, host-directed therapy, immunomodulation, omega-3 fatty acids, real-world study, sepsis

## Abstract

**Objective:**

Sepsis remains a leading cause of intensive care unit (ICU) mortality worldwide, characterized by dysregulated inflammation and immune dysfunction mechanisms also central to many neglected tropical diseases. Omega-3 fatty acids (Ω-3 FAs) possess potent anti-inflammatory and immunomodulatory properties that may improve survival outcomes in such conditions. This retrospective real-world study evaluated the impact of Ω-3 FA supplementation on ICU mortality among patients with sepsis and identified prognostic factors influencing therapeutic efficacy.

**Methods:**

Patients admitted with sepsis to the ICU of Shenzhen People’s Hospital between December 2016 and July 2019 were retrospectively analyzed. Propensity score matching (PSM) was applied at a 1:2 ratio between Ω-3 FA-treated and control groups using covariates including age, sex, diagnosis, norepinephrine (NE) requirement, hemofiltration (HF), C-reactive protein (CRP), and lymphocyte count. Logistic regression and inverse probability of treatment weighting (IPTW) were performed to determine the independent effect of Ω-3 FAs on mortality.

**Results:**

A total of 633 patients were included (Ω-3 FA group, *n* = 211; control, *n* = 422). The unadjusted mortality rate was 32.7% in the Ω-3 FA group and 24.6% in controls (*p* = 0.032). Univariate analysis showed a weak protective effect of Ω-3 FAs (HR = 0.74, 95% CI: 0.54–1.02, *p* = 0.062). After adjusting for age, HF and NE requirements, CRP, lymphocyte count, Sequential Organ Failure Assessment (SOFA) score, and abdominal infection, Ω-3 FAs demonstrated a significant protective effect (HR = 0.60, 95% CI: 0.43–0.83, *p* = 0.003). Kaplan–Meier analysis confirmed improved survival in the Ω-3 FA group (*p* = 0.038). Advanced age, elevated CRP, and higher NE dependence were identified as factors that negatively modulated Ω-3 FA efficacy.

**Conclusion:**

Omega-3 fatty acid supplementation was associated with significantly reduced adjusted ICU mortality in sepsis, underscoring its host-directed immunomodulatory properties. These findings highlight the translational potential of Ω-3 FAs as adjunct therapeutic agents in sepsis and other infection-associated inflammatory disorders, supporting further drug development toward host-directed treatments for neglected tropical diseases.

## Background

Sepsis is a life-threatening clinical syndrome resulting from a dysregulated host response to infection, leading to widespread inflammation, tissue damage, and multi-organ failure (Cao et al., 2023; Baddam and Burns, 2025). Despite major advances in antibiotic therapy, hemodynamic support, and critical care technologies, the mortality rate associated with sepsis remains unacceptably high, ranging from 25% to 40% in intensive care units (ICUs) worldwide (Waxman et al., 2005; La Via et al., 2025). Current therapeutic interventions primarily aim to eradicate infection and maintain organ function through modalities such as mechanical ventilation (MV), continuous renal replacement therapy (CRRT), and vasopressor administration—most commonly norepinephrine (NE) to sustain blood pressure (La Via et al., 2025). However, these approaches offer limited impact on the underlying immunopathology of sepsis, which is characterized by uncontrolled inflammation, immune exhaustion, and metabolic dysregulation. Conventional immunomodulatory therapies including corticosteroids, cytokine antagonists, complement or coagulation regulators, and intravenous immunoglobulins have demonstrated inconsistent or modest benefits, emphasizing the need for alternative host-directed therapeutic strategies that can modulate immune responses rather than merely suppress infection (Jain et al., 2024). Omega-3 fatty acids (Ω-3 FAs), a subclass of polyunsaturated fatty acids (PUFAs), have gained increasing attention for their anti-inflammatory and immunoresolving properties (Jerab et al., 2025). These bioactive lipids are precursors of specialized pro-resolving mediators such as resolving, protections, and margins, which actively terminate inflammation, enhance macrophage-mediated clearance, and promote tissue repair. Experimental and clinical studies have suggested that Ω-3 FA supplementation may improve immune homeostasis, reduce pro-inflammatory cytokine release, and attenuate organ injury in sepsis. For instance, a pilot randomized trial in septic patients with intestinal dysfunction found that fish-oil–based Ω-3 supplementation improved T helper/inducer and CD4/CD8 lymphocyte ratios and reduced 28-day mortality (12.5% vs 41.7%) compared with standard care (Jerab et al., 2025). Nevertheless, the therapeutic efficacy of Ω-3 FAs in sepsis remains controversial. Meta-analyses of randomized controlled trials have shown conflicting results: an earlier analysis of 17 RCTs (1239 patients) reported a non-significant mortality benefit (RR ≈ 0.85, 95% CI 0.71–1.03) but reduction in ICU-length of stay and duration of mechanical ventilation (Lu et al., 2017). A more recent meta-analysis of 20 RCTs (1514 patients) found a significant association between Ω-3 supplementation and reduced mortality (RR = 0.82, 95% CI 0.69–0.97) especially in patients with gastrointestinal dysfunction (Wang et al., 2020; Li et al., 2024). Importantly, evidence derived from randomized trials may not fully capture the complexity of routine clinical practice. Real-world studies provide a valuable complement by reflecting heterogeneous patient populations, treatment variations, and dynamic clinical decision-making. Therefore, this retrospective real-world study was designed to evaluate the impact of Ω-3 FA supplementation on ICU mortality in patients with sepsis. The findings aim to generate clinically meaningful evidence on the host-directed immunomodulatory role of Ω-3 FAs and support their potential development as adjunct therapeutic agents for systemic inflammatory and infection-associated disorders.

## Materials and methods

The study was conducted in accordance with the ethical principles outlined in the Declaration of Helsinki and complied with relevant Chinese regulations. And the study was authorized by the Medical Department of Shenzhen People’s Hospital and approved by the hospital’s Ethics Committee, with the serial number LL-KY-2023207-01. The Ethics Committee granted exemption from obtaining signed informed consent forms for this study. Patient information was obtained through the hospital’s information system for retrospective real-world. The research was conducted in the critical care department of the hospital, which had a capacity of 22 beds.

### Patient selection and eligibility criteria

All patients admitted to the Department of Critical Care Medicine between December 1, 2016, and June 30, 2019, were retrospectively screened according to the diagnostic standards outlined in the Third International Consensus Definitions for Sepsis and Septic Shock (Sepsis-3) (Evans et al., 2021). Eligible participants were those who fulfilled the diagnostic criteria for sepsis, defined as a confirmed or clinically suspected infection accompanied by infection-induced organ dysfunction, indicated by an increase in the Sequential Organ Failure Assessment (SOFA) score of ≥ 2. The site of infection was determined through a comprehensive review of the primary discharge diagnosis, chest radiography, abdominal ultrasonography, and microbiological culture results. Circulatory compromise was defined by the requirement for norepinephrine (NE) infusion as a vasoactive agent, while the need for mechanical ventilation (MV) indicated respiratory failure. Acute kidney injury (AKI) was identified by elevated serum creatinine levels or the requirement for hemofiltration (HF). Hematologic dysfunction was characterized by a platelet count ≤ 100 × 10^9^/L, consistent with coagulopathy. Patients were excluded if they did not meet the diagnostic criteria for infection or if evidence of infection was present without any organ dysfunction.

### Interventions and clinical management

Upon admission to the intensive care unit (ICU), specimens including blood, airway secretions, and drainage fluids were collected for microbial culture to identify infectious pathogens. Empirical antibiotic therapy was initiated based on clinical judgment and subsequently adjusted according to culture and sensitivity results. Patients presenting with septic shock underwent fluid resuscitation for hemodynamic stabilization; intravenous norepinephrine (NE) was administered when mean arterial pressure could not be maintained through fluid therapy alone. Oxygen supplementation was provided to patients with respiratory failure, and mechanical ventilation (MV) was implemented when necessary. For patients who developed acute kidney injury (AKI), continuous hemofiltration (HF) was performed at the discretion of the attending physician. Proton pump inhibitors were administered prophylactically to prevent stress-related mucosal damage. Acute hyperglycemia was managed through continuous intravenous insulin infusion to maintain blood glucose levels within a target range of 8–10 mmol/L. Nutritional support included a high-calorie diet providing approximately 20 kcal/kg within the first week of ICU admission. Omega-3 fatty acid (Ω-3 FA) supplementation was administered as an intravenous 10% lipid emulsion (100 mL containing 10 g refined fish oil; Fresenius Kabi SSPC, China). The emulsion was incorporated into parenteral nutrition and infused once daily. Ω-3 FA administration did not follow a fixed institutional protocol; initiation and continuation were determined by the treating ICU physician based on clinical judgment. Treatment was typically initiated early during ICU admission once nutritional support was established and continued throughout the ICU stay as tolerated. Treatment adherence was confirmed through daily medication and nutrition administration records.

### Clinical and laboratory parameters

Demographic characteristics (age and gender), type and extent of organ failure, and organ support interventions were recorded for all patients. Clinical and biochemical indices of organ function and systemic inflammation including white blood cell count, neutrophil and lymphocyte counts, procalcitonin, and C-reactive protein (CRP) were collected from electronic medical records. Outcome measures included ICU mortality (primary endpoint), duration of mechanical ventilation (hours), and ICU length of stay (days). Patients requiring intravenous NE for hemodynamic support were categorized as “NE needed,” those requiring MV for respiratory failure were categorized as “MV needed,” and those undergoing HF for AKI were categorized as “HF needed.”

### Statistical analysis

Continuous variables were expressed as mean ± standard deviation (SD) for normally distributed data and as medians with interquartile ranges (IQR) for non-normally distributed data. Between-group comparisons were performed using the student’s *t*-test or Mann–Whitney *U*-test, as appropriate. Categorical variables were summarized as frequencies and percentages and compared using the chi-square test. Patients were categorized into two groups based on whether they received intravenous omega-3 fatty acid (Ω-3 FA) supplementation. Covariates influencing sepsis prognosis identified from prior studies (Mendoza et al., 2022; Kim et al., 2024; Zhang et al., 2024) included age, organ failure, organ support measures (norepinephrine use, hemofiltration), comorbidities, lymphocyte count, and Sequential Organ Failure Assessment (SOFA) score. Additional covariates incorporated into the model included gender, primary diagnosis (pneumonia, abdominal infection, cholecystitis, urinary tract infection, or sepsis), C-reactive protein (CRP), and lymphocyte count.

To reduce confounding, propensity score matching (PSM) was applied to balance baseline characteristics between the Ω-3 FA group and the control group at a 1:2 ratio using optimal matching with a caliper width of 0.05 of the standard deviation of the logit of the propensity score. Propensity scores were estimated via logistic regression, and the MatchIt package in *R* was used for implementation. The quality of matching was assessed using standardized mean differences, linear models for continuous variables, and logistic models for binary variables. Cluster-robust standard errors and 95% confidence intervals (CIs) were computed using the vcovCL() function from the *sandwich* package. To further minimize bias, propensity score (PS) matching was performed using two approaches, designated as PS_0_ and PS_1_, to assess comparability and estimate treatment effects between the Ω-3 FA and control groups.PS_0_ matching included covariates such as age, abdominal infection, HF requirement, NE requirement, CRP, lymphocyte count, and SOFA score.PS1 matching incorporated the confounding factors corresponding to each clinical indicator. To further control for residual confounding, Inverse Probability of Treatment Weighting (IPTW) was performed using stabilized weights. Average Treatment Effect (ATE), Average Treatment Effect on the Treated (ATT), and Average Treatment Effect on the Control (ATC) were estimated. Weighted analyses were conducted using svyglm() for continuous and binary outcomes and coxph() for time-dependent survival outcomes. Missing covariate data were addressed using multiple imputation. For categorical variables, missing values were multiply imputed, while for continuous variables, imputation was performed using distribution-appropriate estimates (mean for normally distributed variables and median for skewed variables). Pooled estimates were obtained across more than five imputed datasets. Nonlinear relationships between covariates and outcomes were examined using Generalized Additive Models (GAMs), with the optimal degree of smoothness selected by minimizing the generalized cross-validation (GCV) score. The optimal model form (GAM or Generalized Linear Model [GLM]) was subsequently used for the final analyses. The optimal model structure (GAM or Generalized Linear Model, GLM) was then selected for subsequent analyses. Survival analysis was conducted using Kaplan—Meier curves. To further investigated the effect of Omega-3 FA on clinical outcomes, death was considered as the endpoint, and the length of ICU stay was used as the time parameter. The groups were categorized based on the use of Omega-3 FA, and Kaplan-Meier (KM) curves were plotted. Differences between survival curves were compared using the log-rank test, and multivariable Cox proportional hazards models were employed to adjust for baseline differences. All statistical analyses were performed using EmpowerStats version 4.0 (X&Y Solutions Inc., Boston, MA, USA) and R software version 4.0.4 (R Foundation for Statistical Computing, Vienna, Austria). A *p*-value<0.05 was considered statistically significant.

## Results

**Figure 1:**
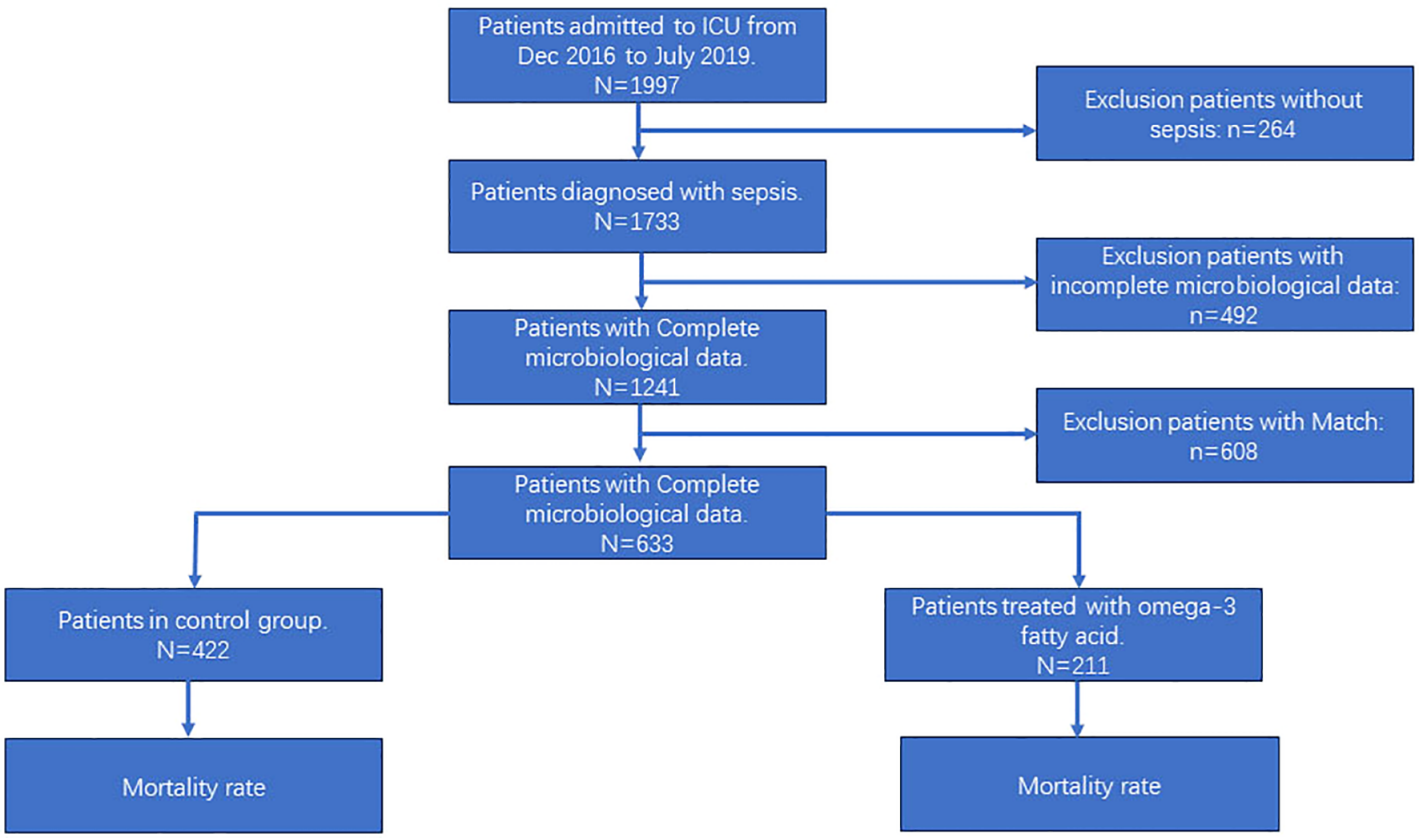
Study cohort flowchart. ICU, intensive care unit.

From December 2016 to July 2019, a total of 1997 patients were admitted to the Department of Intensive Care Medicine at Shenzhen People’s Hospital. Among them, according to inclusion criteria and exclusion criteria, 1733 patients were included in the study. Of these patients, 1013 patients were male and 720 patients were female, with an average age of 61.76 years old,382 patients died, resulting in a mortality rate of 22.04% (refer to **Supplementary Table 1**). 492 patients with missing microbiological test were excluded, and 1241 patients included in the study. Matching was per-formed with the use of 1:2, a total of 633 patients were obtained. Among these, 211 cases were in the treatment group with Omega-3 fatty acid, and 422 cases were in the control group. The patient enrolment was illustrated in **Figure 1** comparative analysis of patient indicators between the two groups showed that the treatment group had a significantly higher mean age, a higher proportion of patients with abdominal infections, and a higher proportion of patients requiring hemofiltration therapy and norepinephrine to maintain blood pressure, C-reactive protein (CRP) levels increased and lymphocyte counts decreased, with higher Sequential Organ Failure assessment (SOFA) scores. The distribution of variables in the two groups is presented in **Table 1**.

**Table 1:** Distribution of baseline covariates by treatment.

Variables	Control group	Omega-3 FA group	P value
Case	422	211	-
Gender			0.0952
Male, case %	260 (61.6)	145 (68.7)	
Female, case %	162 (38.4)	66 (31.3)	
AGE(y, M ± SD)	(422) 62.83 ± 20.12	(211) 66.54 ± 18.22	0.0246
Pneumonia, case %			0.3037
No	143 (33.9)	81 (38.4)	
Yes	279 (66.1)	130 (61.6)	
Abdominal infection, case %			0.0384
No	403 (95.5)	192 (91)	
Yes	19 (4.5)	19 (9)	
Biliary infection, case %			0.9103
No	416 (98.6)	207 (98.1)	
Yes	6 (1.4)	4 (1.9)	
Urinary infection, case %			0.8739
No	418 (99.1)	210 (99.5)	
Yes	4 (0.9)	1 (0.5)	
Septicemia, case %			0.0777
No	343 (81.3)	158 (74.9)	
Yes	79 (18.7)	53 (25.1)	
MV needed, case %			0.1496
No	135 (32)	55 (26.1)	
Yes	287 (68)	156 (73.9)	
HF needed, case %			0.0121
No	323 (76.5)	141 (66.8)	
Yes	99 (23.5)	70 (33.2)	
NE needed, case %			0.035
No	229 (54.3)	95 (45)	
Yes	193 (45.7)	116 (55)	
WBC (x10^9^/L,M ± SD)	(422) 14.27 ± 9.41	(211) 13.61 ± 9.15	0.401
PCT (ng/ml, median)	1.58 (0.30, 11.28)	1.90 (0.49, 10.18)	0.364
CRP(mg/L,M ± SD)	(422) 105.64 ± 89.94	(211) 123.49 ± 94.31	0.021
Lyphocyte counts (/ul, M ± SD)	(422) 1.13 ± 1.01	(211) 0.95 ± 0.98	0.037
SOFA (M ± SD)	(422) 9.87 ± 4.27	(211) 11.04 ± 4.44	0.001

FA, fatty acids; MV, mechanical ventilation; HF, hemofiltration; NE, norepinephrine; WBC, white blood cell count; PCT, procalcitonin; CRP, C reactive protein; SOFA, sequence organ failure assessment.

### Clinical outcome

In total, 69 deaths (32.7%) occurred in the Omega-3 fatty acid (Ω-3 FA) treatment group and 104 deaths (24.6%) in the control group, indicating a significantly higher crude mortality in the Ω-3 FA group (*χ²* = 4.60, *p* = 0.032). Both the duration of mechanical ventilation (MV, hours) and the length of ICU stay (days) were significantly longer among patients receiving Ω-3 FA compared with the control group (*p* < 0.001 for both; **Supplementary Table 2**). These findings suggest that patients treated with Ω-3 FA were clinically more severe at baseline, necessitating longer ICU management and ventilatory support. These crude differences likely reflect baseline clinical severity, as patients receiving ω-3 FA supplementation presented with significantly higher age, greater need for hemofiltration and norepinephrine support, elevated CRP, lower lymphocyte counts, and higher SOFA scores at admission, all of which are strong predictors of mortality.

### Univariate and multivariate analysis

Univariate logistic regression analysis indicated that Ω-3 FA therapy exhibited a potential but statistically nonsignificant protective trend against sepsis-related mortality (*p* > 0.05). In contrast, several clinical and biochemical variables demonstrated significant associations with mortality risk. Specifically, mortality increased by 1.4% with each additional year of age (*p* = 0.002). Patients requiring norepinephrine (NE) to maintain hemodynamic stability exhibited a 61% higher mortality risk compared with those not requiring vasopressor support (*p* = 0.006). Similarly, for every 1 mg/L increase in C-reactive protein (CRP) concentration, mortality rose by 0.2% (*p* = 0.019). Moreover, each one-point increment in the SOFA score corresponded to a 12.7% increase in mortality (*p* < 0.001) (**Table 2****).** After adjusting for confounding covariates—including age, requirement for hemofiltration (HF), NE use, CRP level, lymphocyte count, SOFA score, and abdominal infection—the multivariate logistic regression revealed that the protective effect of Ω-3 FA became statistically significant (HR = 0.600, 95% CI 0.428–0.828; *p* = 0.003) (**Supplementary Tables 3**, **4****).**

**Table 2:** Univariate analysis for treatment and covariates.

Exposure	Statistics	Death
Omega-3 FA supplement		
No	422 (66.667%)	1.0
Yes	211 (33.333%)	0.740 (0.539, 1.015) 0.0616
Gender		
Male	405 (63.981%)	1.0
Female	228 (36.019%)	1.364 (0.995, 1.869) 0.0534
AGE	64.070 ± 19.570	1.014 (1.005, 1.023) 0.0018
Pneumonia		
No	224 (35.387%)	1.0
Yes	409 (64.613%)	0.998 (0.732, 1.361) 0.9907
Abdominal infection		
No	595 (93.997%)	1.0
Yes	38 (6.003%)	0.995 (0.465, 2.129) 0.9890
Biliary infection		
No	623 (98.420%)	1.0
Yes	10 (1.580%)	0.925 (0.229, 3.734) 0.9131
Urinary infection		
No	628 (99.210%)	1.0
Yes	5 (0.790%)	1.890 (0.467, 7.641) 0.3718
Septicemia		
No	501 (79.147%)	1.0
Yes	132 (20.853%)	1.011 (0.728, 1.405) 0.9468
MV needed		
No	190 (30.016%)	1.0
Yes	443 (69.984%)	1.176 (0.755, 1.831) 0.4732
HF needed		
No	464 (73.302%)	1.0
Yes	169 (26.698%)	1.296 (0.955, 1.757) 0.0960
NE needed		
No	324 (51.185%)	1.0
Yes	309 (48.815%)	1.611 (1.146, 2.264) 0.0060
WBC	14.046 ± 9.321	0.988 (0.971, 1.005) 0.1709
PCT	13.700 ± 30.568	1.006 (0.998, 1.014) 0.1721
CRP	111.588 ± 91.734	1.002 (1.000, 1.003) 0.0189
Lymphocyte counts	1.070 ± 1.001	0.917 (0.776, 1.083) 0.3080
SOFA	10.261 ± 4.361	1.127 (1.089, 1.167) <0.0001

Results in table: β (95%CI) P value / OR (95%CI) *P* value.

FA, fatty acids; HE, hemofiltration; NE, norepinephrine; WBC, white blood cell counts; PCT, procalcitonin; CRP, C-reactive protein; SOFA, sequence organ failure assessment.

Following PS_0_ matching, no significant baseline differences were observed between the treatment and control groups (*p* > 0.05 for all indices). After PS_1_ matching, only abdominal infection remained significantly different between the two groups (*p* = 0.005), while other variables were comparable (*p* > 0.05) (**Table 3****).** Importantly, the protective effect of Ω-3 FA on mortality remained consistent across both PS_0_ and PS_1_ models using different matching algorithms. Before adjusting for all covariates, the crude HR value was 0.597, with 95% CI 0.539-1.015, and the *P* value was 0.0616. When adjusting for all relevant confounders, Ω-3 FA therapy continued to demonstrate a significant protective effect in multivariate models (HR = 0.597, 95% CI 0.430–0.840; *p* = 0.003) (**Supplementary Table 4****).** Under PS_0_ matching, the average treatment effect on the treated (ATT) revealed a hazard ratio (HR) of 0.473 (95% CI 0.338–0.662, *p* < 0.001), indicating that Ω-3 FA significantly reduced mortality among treated patients. The average treatment effect on the controls (ATC) was also significant (HR = 0.565, 95% CI 0.366–0.873, *p* = 0.010), suggesting that patients in the control group would likely have benefited from Ω-3 FA treatment. When evaluating the average treatment effect (ATE) across all patients, Ω-3 FA maintained a significant protective association with mortality (HR = 0.568, 95% CI 0.385–0.838, *p* = 0.004). Similarly, the analysis matched according to PS_1_ confirmed the significant protective effect of Ω-3 FA therapy (*p* < 0.05) (**Table 4****).** The ATT, ATC, and ATE estimates derived using inverse probability of treatment weighting (IPTW) further reinforced the robustness of these findings, consistently indicating a mortality-reducing benefit of Ω-3 FA supplementation (**Table 5****).** The corresponding weight distributions utilized in IPTW are provided in **Supplementary Table 5**.

**Table 3:** Balance report after PS Match (using ATE weights).

Variables	Control group	Omega-3 FA^*^ group	P value
Match PS0			
Age	63.4083 ± 19.8017	64.8289 ± 19.5367	0.3911
CRP	111.4508 ± 91.1213	114.1595 ± 96.4267	0.7346
Lymphocyte counts	1.1151 ± 1.0423	1.1168 ± 1.0087	0.9843
SOFA score	10.5001 ± 4.4985	10.0982 ± 4.1859	0.2674
ps0	0.3331 ± 0.1056	0.3337 ± 0.1073	0.9453
ps1	0.3325 ± 0.0969	0.3338 ± 0.1007	0.8794
HF needed			0.8613
No	0.71	0.72	
Yes	0.29	0.28	
NE needed			0.3197
No	0.49	0.53	
Yes	0.51	0.47	
Abdominal infection			0.8975
No	0.94	0.94	
Yes	0.06	0.06	
Match PS1			
Age	63.9224 ± 19.6981	64.9234 ± 18.9222	0.5364
CRP	112.5213 ± 92.7504	110.0239 ± 93.1505	0.7503
Lymphocyte counts	1.0537 ± 0.9622	1.0508 ± 0.9982	0.9722
SOFA score	10.3206 ± 4.3847	10.0850 ± 4.3506	0.5222
ps0	0.3307 ± 0.1045	0.3415 ± 0.1091	0.2368
ps1	0.3328 ± 0.0979	0.3341 ± 0.1011	0.8770
HF needed			0.8720
No	0.74	0.74	
Yes	0.26	0.26	
NE needed			0.9664
No	0.50	0.51	
Yes	0.50	0.49	
Abdominal infection			0.0053
No	0.95	0.89	
Yes	0.05	0.11	

Omega-3 FA: Omega-3 fatty acid.

**Table 4:** Average therapeutic benefits of the treatment group and the control group after PS0 and PS1 were used to match the two groups.

	Death
Match PS0^†^	
ATT	0.473 (0.338, 0.662) <0.0001
ATC	0.565 (0.366, 0.873) 0.0102
ATE	0.568 (0.385, 0.838) 0.0044
Match PS1^††^	
ATT	0.619 (0.441, 0.868) 0.0055
ATC	0.665 (0.438, 1.009) 0.0550
ATE	0.654 (0.459, 0.933) 0.0193

Results in table: HR (95%CI) P value. Cluster-robust standard errors ^11^ were applied for calculating 95% CI.

^†^Match PS0: propensity score calculated with age, abdominal infection, HF needed, NE needed, CRP, lymphocyte count, and SOFA score.

^††^Match PS1: propensity score calculated by confounders.

Confounders: Age, HF needed, NE needed, CRP(smooth), Lymphocyte counts (smooth), SOFA(smooth). ATT, average treatment effect for treated; ATC: average treatment effect for control; ATE, average treatment effect for all.

**Table 5:** Estimate of treatment effects using IPTW.

IPW using PS0	Death
ATT	Robust CI: 0.580 (0.420, 0.800) 0.0009; Survey Wald CI: 0.580 (0.420, 0.800) 0.0009
ATC	Robust CI: 0.715 (0.507, 1.007) 0.0545; Survey Wald CI: 0.715 (0.507, 1.007) 0.0545
ATE	Robust CI: 0.665 (0.480, 0.921) 0.0140; Survey Wald CI: 0.665 (0.480, 0.921) 0.0140
IPW using PS1	DEATH
ATT	Robust CI: 0.604 (0.438, 0.831) 0.0020; Survey Wald CI: 0.604 (0.438, 0.831) 0.0020
ATC	Robust CI: 0.696 (0.493, 0.983) 0.0398; Survey Wald CI: 0.696 (0.493, 0.983) 0.0398
ATE	Robust CI: 0.664 (0.479, 0.921) 0.0141; Survey Wald CI: 0.664 (0.479, 0.921) 0.0141

Results in table: HR (95%CI) P value. IPTW: inverse probability of treatment weighting using the propensity score.

PS0: propensity score calculated by Age, HF needed, NE needed, CRP(smooth), Lymphocyte counts (smooth), SOFA(smooth), Abdominal infection. Confounders: Age, HF needed, NE needed, CRP(smooth), Lymphocyte counts (smooth), SOFA(smooth). PS1: propensity score calculated by confounders: Age, HF needed, NE needed, CRP(smooth), Lymphocyte counts (smooth), SOFA(smooth).

ATT, average treatment effect for treated; ATC, average treatment effect for control; ATE, average treatment effect for all.

### Survival analysis

To further investigated the effect of Omega-3 FA on clinical outcomes, death was considered as the endpoint, and the length of ICU stay was used as the time parameter.

**Figure 2:**
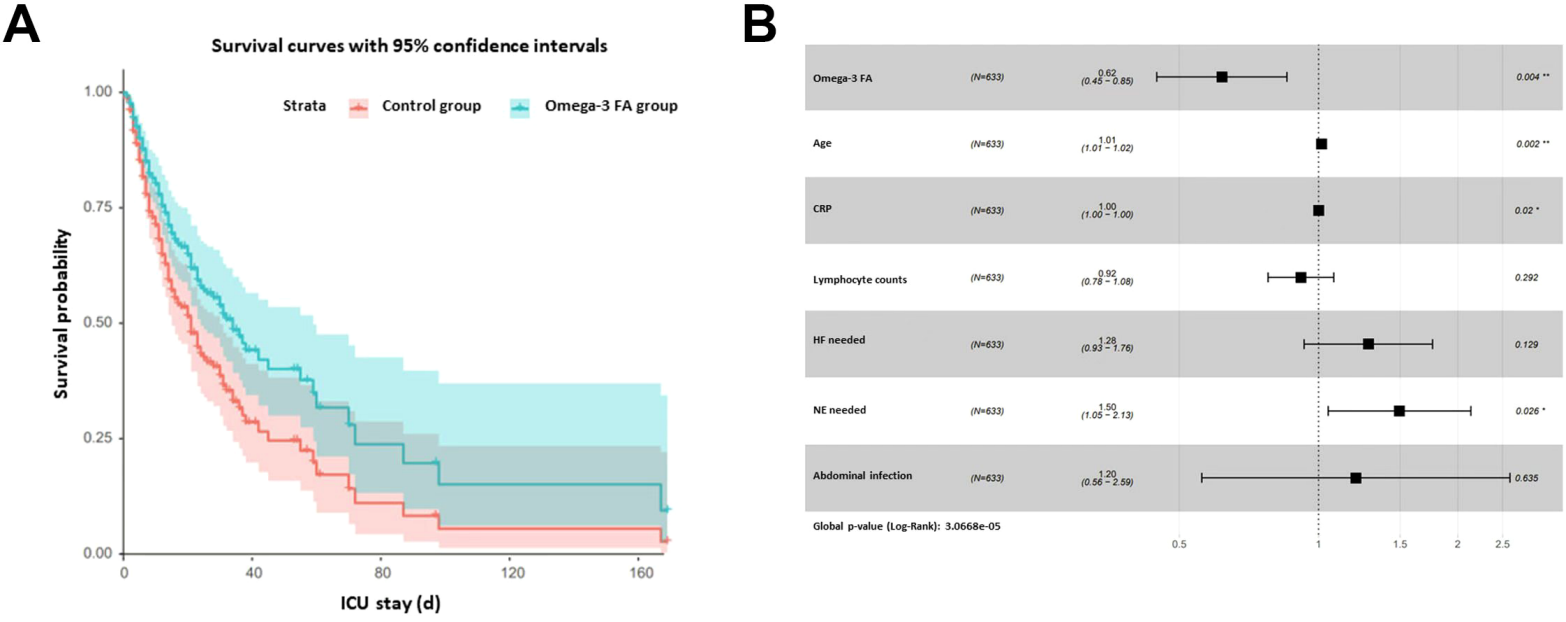
Adjusted Kaplan-Meier curve. **(A)** the KM curve was adjusted for age, abdominal infection, HF requirement, NE requirement, CRP, and lymphocyte count. **(B)** Multivariate analysis indicated that age, CRP, and the NE needed to maintain blood pressure had a negative impact on the effectiveness of Omega-3 FA in sepsis treatment. HF, hemofiltration; NE, norepinephrine; CRP, C-reactive protein.

The groups were categorized based on the use of Omega-3 FA, and the Kaplan-Meier (KM) curves of the two groups were overlapped, indicating no significant difference between them (*p* = 0.058). The KM curve demonstrated that the benefit of Omega-3 FA treatment primarily occurred in the later stages (**Supplementary Figure 1**). Upon comparing the results of the two groups in **Table 1**, the KM curve was adjusted for age, abdominal infection, HF requirement, NE requirement, CRP levels, and lymphocyte count. The adjusted curve showed a statistically significant difference between the two groups (*p* = 0.038), diverging from the KM curve generated by the original data. The adjusted curve suggested that the Omega-3 FA treatment group had better clinical outcomes (**Figure 2A**). Multivariate analysis indicated that age, CRP levels, and the NE needed to maintain blood pressure had a negative impact on the effectiveness of Omega-3 FA in sepsis treatment (**Figure 2B**). The results of multivariate analysis for each factor can be found in **Supplementary Table 6**.

## Discussion

Sepsis is a life-threatening clinical syndrome characterized by a dysregulated host response to infection, leading to profound and potentially fatal organ dysfunction (Kamath et al., 2023). Throughout this process, patients go through various stages, such as excessive inflammatory responses and immunosuppression. Despite active treatment, the mortality rate associated with sepsis is alarmingly high (Rudd et al., 2018). Finding new treatment methods is of significant value for sepsis. Omega-3 fatty acids are considered a potentially effective therapy. The expert consensus in USA recommends that for patients with sepsis who require parenteral nutrition, fish oil fat emulsion can be administered (Mundi et al., 2021). As the human body lacks the enzymes required for their synthesis, Omega-3 fatty acids are considered essential and must be acquired through dietary sources like fish oil (10g, Fresenius Kabi SSPC). The main physiological function of Omega-3 fatty acids is to regulate the body’s inflammatory response, alleviate immune suppression, thereby reducing tissue and organ damage, and improving prognosis (Calder, 2019; Zaloga, 2021; Notz et al., 2022a). The use of polyunsaturated FA in sepsis has a long history but it has been accompanied by significant controversy (Heller et al., 2011). Early randomized controlled trials suggested that fish oil did not confer any protective effects on organ failure in sepsis patients (Wohlmuth et al., 2010). However, other studies had indicated that fish oil may indeed have a beneficial effect on organ failure in sepsis patients (Hall et al., 2015). More and more positive evidences support the use of omega-3 FA in critically ill patients in the ICU (Singer and Calder, 2023). Several clinical studies have reported the efficacy of Omega-3 fatty acids in organ protection in sepsis. FA has been found to benefit various organ functions in sepsis patients. For instance, FA can inhibit excessive apoptosis of cardiomyocytes and improve myocardial damage in sepsis patients (Chen et al., 2023). Additionally, inhibiting the inflammatory response can have a protective effect on cardiomyocytes (Busch et al., 2021).FA can inhibit inflammation, improve lymphocyte immune function and reduce mortality in patients with sepsis induced intestinal failure (Chen et al., 2017).The study also found that indicators of liver function improved in patients with septic liver failure who received fish oil treatment (Badia-Tahull et al., 2015). Furthermore, liver disease induced by intestinal failure can benefit from Omega-3 FA, which act as anti-inflammatory agents and can reverse cholestasis (Bolia and Srivastava, 2019). For patients with acute lung injury, enteral nutrition formula rich inomega-3 PUFAs has positive effects on shortening mechanical ventilation time and reducing mortality (Singer et al., 2006). In sepsis patients with COVID-19, Omega-3 FA have demonstrated anti-inflammatory effects and can significantly reduce mortality (Erdem et al., 2023).Meta-analysis has shown that FA can reduce the 28-day mortality in severe patients (Notz et al., 2022a). However, the studies were mostly clinical trials rather than real-world studies.

To better assess the efficacy of omega-3 fatty acids in real-world medical settings, we conducted a retrospective review of data from 1,733 patients with sepsis, performing a real-world study. After excluding patients without pathogenic microbiome record and using propensity score matching, a total of 633 patients (422 controls and 211 treated) were included in the study. The mortality was 24.64% in the control group and 32.7% in the Omega-3 FA treatment group. The mortality of the treatment group was higher than that of the control group, which was different from previous research results (Chen et al., 2017),and there were significant differences in age, HF needed, NE needed, CRP, lymphocyte count, SOFA score and abdominal infection between the two groups. Although the crude mortality was higher in the ω-3 FA group, this finding reflects marked baseline imbalances rather than treatment harm. Patients receiving ω-3 FA supplementation were older and exhibited greater disease severity, including higher SOFA scores, more frequent hemofiltration and norepinephrine use, and higher inflammatory burden at ICU admission. These factors are independently associated with increased mortality in sepsis and likely biased the unadjusted comparison. After rigorous adjustment using multivariable models, propensity score matching, and IPTW to balance these prognostic indicators, ω-3 FAs consistently demonstrated a significant protective association with mortality, indicating that the crude unfavorable pattern primarily resulted from confounding. The study is a retrospective real-world study with data from hospital medical records. It is difficult to fully control and adjust confounding factors for patients entering the study based on their actual conditions. Previous studies have shown that the mortality of sepsis patients was significantly correlated with age, sofa score, infection site and other factors (Kim et al., 2024; Zhang et al., 2024).In the intensive care unit (ICU), critically ill patients necessitate increased equipment or medication support, indicating a more severe condition (Frank et al., 2021).One study identified age, Sequential Organ Failure Assessment (SOFA) score, use of vasopressors, and mechanical ventilation as independent risk factors for mortality (Liu et al., 2022).The above factors may affect the efficacy of Omega-3 fatty acids. So, we further performed univariate logistic regression analysis, and the statistical results showed that multiple factors, such as age, CRP, NE needed, etc, were significantly correlated with the mortality of patients with sepsis. After adjusting for age, HF needed, NE needed, CRP, lymphocyte count, SOFA score, and presence or absence of abdominal infection, multivariate logistic regression analysis showed that the protective effect of Omega-3 FA became apparent (HR 0.600, 95% CI 0.428, 0.828; *p* = 0.003). After adjustment, ω-3 FA supplementation was associated with a 35–45% relative reduction in mortality risk, which represents a clinically meaningful effect size in the context of sepsis, where even modest survival improvements are highly impactful. In addition, we found that age, CRP levels, and NE needed negatively affect the effectiveness of Omega-3 FA in treating sepsis. Age, CRP, and NE needed are key factors affecting the efficacy of Omega-3 FA.

According to the results of univariate logistic regression analysis and multivariate logistic regression analysis, we optimized the matching model in the follow-up study, and adopted IPTW to control confounding factors. The results showed that Omega-3 fatty acids had a good effect on reducing the mortality of sepsis, and the clinical consistency was good. After adjusting the survival curve with the aforementioned indicators, the survival analysis results show that Omega-3 fatty acids have a protective effect on the prognosis of sepsis. Previous clinical studies have mostly come from randomized controlled trials (RCTS), which tend to be conducted in specific populations, which may lead to limited application of the findings in practice. At the same time, there are many factors affecting the prognosis of sepsis, and it is difficult to explain the effect of single therapy only by direct comparison. Our study is the first real world study of Omega-3 fatty acids for sepsis; The significance of this study is the combination of Propensity Score Matching (PSM) and Inverse Probability of Treatment Weighting (IPTW). By controlling confounding variables and balancing the difference in baseline characteristics between different groups, the effect of randomized controlled trials was simulated to a certain extent, so as to estimate the treatment effect more accurately and obtain the average efficacy of treatment with Omega-3 fatty acids in the treatment group and the hypothetical control group. The study results suggest that the protective effect of Omega-3 fatty acids on mortality is consistent. It can better guide the clinical application of Omega-3 fatty acids. Therefore, based on the mechanism and clinical evidence, it was evident that Omega-3 FA had beneficial effects on inflammation reduction, organ protection, and cellular immunity in sepsis (Rosenthal et al., 2021). Although this study was conducted in a single-center retrospective real-world setting, this design also allowed evaluation of ω-3 FA therapy under routine clinical practice conditions, enhancing its clinical relevance. Because treatment was physician-directed rather than protocol-based, patients receiving ω-3 FA tended to be clinically more severe at baseline. While this inevitably introduced treatment-indication bias, it also provided an opportunity to assess potential benefit in high-risk patients. Advanced analytical approaches including multivariable adjustment, propensity score matching, and IPTW were applied to minimize confounding, and the consistency of results across multiple models strengthens confidence in the observed association, although causality cannot be definitively inferred. ω-3 FA administration occurred as part of parenteral nutrition, meaning treatment timing, duration, and cumulative dose varied naturally across patients. Rather than being a limitation alone, this reflects real-world heterogeneity and demonstrates applicability of findings to diverse ICU scenarios. Similarly, while inflammatory biomarkers such as cytokines were unavailable, routinely monitored indicators (CRP, PCT, WBC) still supported clinically meaningful biological plausibility. Immortal-time bias cannot be fully excluded because ω-3 FA typically began after stabilization; however, this reflects real-world constraints of nutrition initiation rather than methodological weakness. Importantly, these real-world observations generated a coherent signal suggesting that ω-3 FA use is associated with improved adjusted survival despite the treated group being initially sicker, highlighting meaningful clinical potential. Moving forward, multicenter prospective studies incorporating standardized treatment protocols, time-dependent exposure modeling, detailed nutritional assessment, and immune biomarker profiling will be valuable to validate these findings, better characterize biological mechanisms, and refine patient selection. Such studies will help translate this promising real-world evidence into optimized therapeutic strategies.

## Conclusion

This retrospective analysis explored the link between ω-3 fatty acid supplementation and outcomes in sepsis patients. Omega-3 fatty acid supplementation was associated with reduced adjusted ICU mortality in sepsis; the study demonstrates that intravenous administration of ω-3 fatty acids confers a protective effect on mortality among ICU patients with sepsis after adjustment for confounding factors. These findings support the immunomodulatory and pro-resolving potential of ω-3 FA and justify their inclusion as an adjunctive therapeutic strategy in sepsis management. this conclusion was tempered by two key factors: the inherent limitations of the retrospective design, which preclude ruling out unmeasured confounding variables, and the higher crude mortality rate observed in the treatment group. Further confirmation requires well-powered prospective randomized controlled trials to clarify the efficacy, optimal dosage, and administration timing of ω-3 fatty acids for sepsis.

## Data Availability

The datasets presented in this study can be found in online repositories. The names of the repository/repositories and accession number(s) can be found in the article/**Supplementary Material**.
