# Supplementary figures and images for: Omega-3 fatty acids as host-directed immunomodulatory therapeutics in sepsis: real-world evidence supporting drug development potential for systemic inflammatory diseases
Source: Front Cell Infect Microbiol. 2026 Jan 26;15:1738204. doi: 10.3389/fcimb.2025.1738204 (PMC12883643; doi:10.3389/fcimb.2025.1738204)

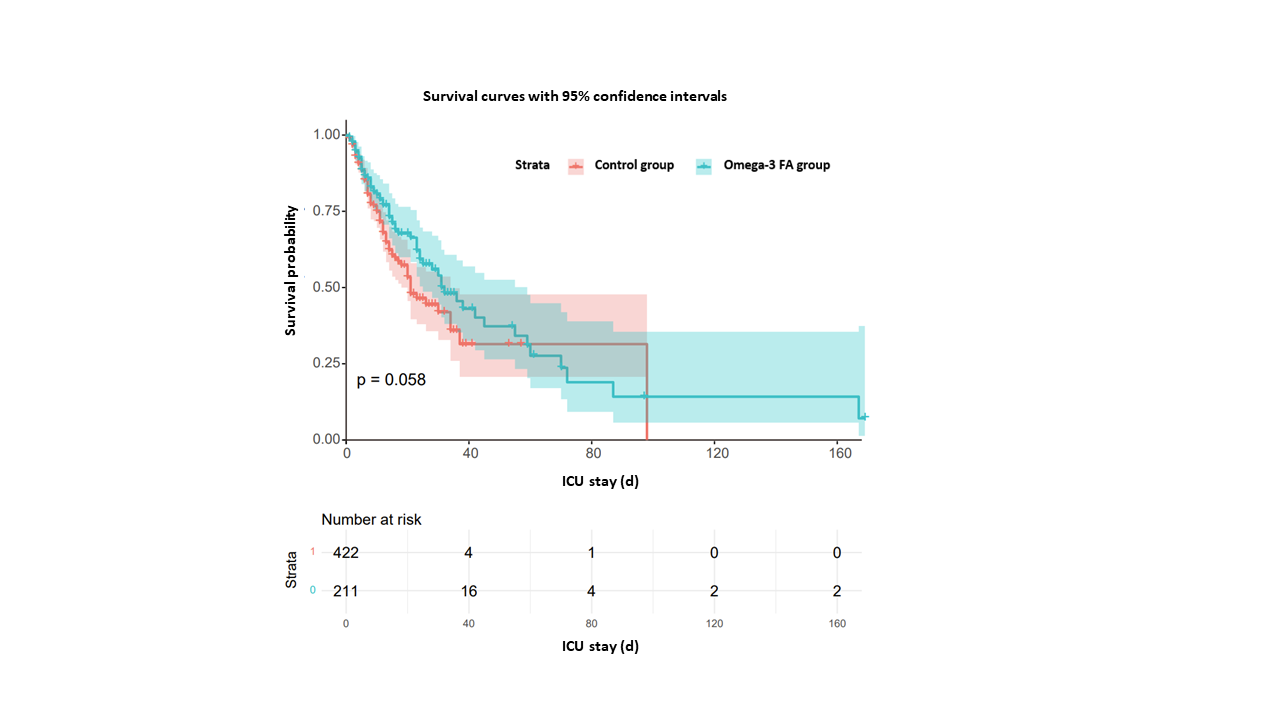

Supplement: Supplementary file 1 [file Image1.tif]
