# Supplementary material for: Omega-3 fatty acids as host-directed immunomodulatory therapeutics in sepsis: real-world evidence supporting drug development potential for systemic inflammatory diseases
Source: Front Cell Infect Microbiol. 2026 Jan 26;15:1738204. doi: 10.3389/fcimb.2025.1738204 (PMC12883643; doi:10.3389/fcimb.2025.1738204)
Supplement: Supplementary file 2 [file DataSheet1.docx]

Supplementary tables

Supplementary table -1 Total Study Population Description

|  | N | Statistics |
| --- | --- | --- |
| Age (y, Mean±SD) | 1733 | 61.76 ± 20.34 |
| Gender |  |  |
| Male (%) | 1013 | 58.45 |
| Female (%) | 720 | 41.55 |
| **Diagnosis** |  |  |
| Pneumonia |  |  |
| No (%) | 635 | 36.64 |
| Yes (%) | 1098 | 63.36 |
| Abdominal infection |  |  |
| No (%) | 1650 | 95.21 |
| Yes (%) | 83 | 4.79 |
| Biliatry infection |  |  |
| No (%) | 1707 | 98.50 |
| Yes (%) | 26 | 1.50 |
| Urinary infection |  |  |
| No (%) | 1725 | 99.54 |
| Yes (%) | 8 | 0.46 |
| Septicemia |  |  |
| No (%) | 1513 | 87.31 |
| Yes (%) | 220 | 12.69 |
| **Organ dysfunction assessment** |  |  |
| MV needed |  |  |
| No (%) | 666 | 38.43 |
| Yes (%) | 1067 | 61.57 |
| HF needed |  |  |
| No (%) | 1388 | 80.09 |
| Yes (%) | 345 | 19.91 |
| NE needed |  |  |
| No (%) | 1067 | (61.570%) |
| Yes (%) | 666 | 38.43 |
| Plateletless |  |  |
| No (%) | 1412 | 77.96 |
| Yes (%) | 612 | 22.04 |
| Hyperbilirubinemia |  |  |
| No (%) | 1412 | 81.48 |
| Yes (%) | 321 | 18.52 |
| SOFA (Mean±SD) | 1733 | 9.08±4.25 |
| **Clinical outcome** |  |  |
| Death |  |  |
| No (%) | 1351 | 77.96 |
| Yes (%) | 382 | 22.04 |
| ICU stay (d, Median(Q1,Q3)) | 1733 | 5.00 (2.00, 10.00) |
| MV duration (h, Median(Q1,Q3)) | 1191 | 46.00(15.00,148.00) |

Note: MV: mechanical ventilation. HE: hemofiltration. NE: norepinephrine. SOFA: sequence organ failure assessment. ICU: intensive care unit.

Supplementary table-2 Regression coefficient of treatment and its change after adjust for each covariate

| Covariates | DEATH |
| --- | --- |
| Total | -0.3017 |
| Gender | -0.2827 |
| Age | -0.3492 |
| Pneumonia | -0.3045 |
| Abdominal infection | -0.3040 |
| Biliatry infection | -0.3022 |
| Urinary infection | -0.3007 |
| Septicemia | -0.3032 |
| MV needed | -0.3008 |
| HF needed | -0.3644 |
| NE needed | -0.3667 |
| WBC | -0.3016 |
| PCT | -0.6069 |
| CRP | -0.3301 |
| Lymphoctye count | -0.3099 |
| SOFA score | -0.4948 |
| MV duration | -0.0933 |

Note: MV: mechanical ventilation. HE: hemofiltration. NE: norepinephrine. SOFA: sequence organ failure assessment. WBC: white blood cell counts. PCT: procalcitonin. CRP: C-reactive protein.

Supplementary table-3 Associations of treatment with outcomes

| Model | DEATH |
| --- | --- |
| Crude | 0.740 (0.539, 1.015) 0.0616 |
| Adjust for all covariates | 0.600 (0.428, 0.840) 0.0030 |
| Adjust for PS0 | 0.597 (0.430, 0.828) 0.0020 |
| Adjust PS0(smooth) | 0.583 (0.420, 0.811) 0.0013 |
| Adjust for confounders | 0.600 (0.428, 0.840) 0.0030 |
| Adjust for PS1 | 0.597 (0.430, 0.828) 0.0020 |
| Adjust PS1(smooth) | 0.589 (0.424, 0.817) 0.0015 |

Note:
Results in table: HR (95%CI) P value
Note for models: 
All covariates: Age, HF needed, NE needed, CRP(smooth), Lymphocyte counts (smooth), SOFA(smooth), Abdominal infection. 
PS0: propensity score calculated by Age, HF needed, NE needed, CRP(smooth), Lymphocyte counts (smooth), SOFA(smooth), Abdominal infection.
Confounders: Age, HF needed, NE needed, CRP(smooth), Lymphocyte counts (smooth), SOFA(smooth).
PS1: propensity score calculated by confounders: Age, HF needed, NE needed, CRP(smooth), Lymphocyte counts (smooth), SOFA(smooth).

Supplementary table-4 Balance report after PS Match (using ATE weights)

| Variables | Control group | Omega-3 FA^*^ group | P value |
| --- | --- | --- | --- |
| Match PS0 |  |  |  |
| Age | 63.4083 ± 19.8017 | 64.8289 ± 19.5367 | 0.3911 |
| CRP | 111.4508 ± 91.1213 | 114.1595 ± 96.4267 | 0.7346 |
| Lymphocyte counts | 1.1151 ± 1.0423 | 1.1168 ± 1.0087 | 0.9843 |
| SOFA score | 10.5001 ± 4.4985 | 10.0982 ± 4.1859 | 0.2674 |
| ps0 | 0.3331 ± 0.1056 | 0.3337 ± 0.1073 | 0.9453 |
| ps1 | 0.3325 ± 0.0969 | 0.3338 ± 0.1007 | 0.8794 |
| HF needed |  |  | 0.8613 |
| No | 0.71 | 0.72 |  |
| Yes | 0.29 | 0.28 |  |
| NE needed |  |  | 0.3197 |
| No | 0.49 | 0.53 |  |
| Yes | 0.51 | 0.47 |  |
| Abdominal infection |  |  | 0.8975 |
| No | 0.94 | 0.94 |  |
| Yes | 0.06 | 0.06 |  |
| Match PS1 |  |  |  |
| Age | 63.9224 ± 19.6981 | 64.9234 ± 18.9222 | 0.5364 |
| CRP | 112.5213 ± 92.7504 | 110.0239 ± 93.1505 | 0.7503 |
| Lymphocyte counts | 1.0537 ± 0.9622 | 1.0508 ± 0.9982 | 0.9722 |
| SOFA score | 10.3206 ± 4.3847 | 10.0850 ± 4.3506 | 0.5222 |
| ps0 | 0.3307 ± 0.1045 | 0.3415 ± 0.1091 | 0.2368 |
| ps1 | 0.3328 ± 0.0979 | 0.3341 ± 0.1011 | 0.8770 |
| HF needed |  |  | 0.8720 |
| No | 0.74 | 0.74 |  |
| Yes | 0.26 | 0.26 |  |
| NE needed |  |  | 0.9664 |
| No | 0.50 | 0.51 |  |
| Yes | 0.50 | 0.49 |  |
| Abdominal infection |  |  | 0.0053 |
| No | 0.95 | 0.89 |  |
| Yes | 0.05 | 0.11 |  |

Note: Omega-3 FA: Omega-3 fatty acid.

Supplementary table-5 Estimate of treatment effects using IPTW

| IPW using PS0 | DEATH |
| --- | --- |
| ATT | Robust CI: 0.580 (0.420, 0.800) 0.0009;  Survey Wald CI: 0.580 (0.420, 0.800) 0.0009 |
| ATC | Robust CI: 0.715 (0.507, 1.007) 0.0545;  Survey Wald CI: 0.715 (0.507, 1.007) 0.0545 |
| ATE | Robust CI: 0.665 (0.480, 0.921) 0.0140;  Survey Wald CI: 0.665 (0.480, 0.921) 0.0140 |
| IPW using PS1 | DEATH |
| ATT | Robust CI: 0.604 (0.438, 0.831) 0.0020;  Survey Wald CI: 0.604 (0.438, 0.831) 0.0020 |
| ATC | Robust CI: 0.696 (0.493, 0.983) 0.0398;  Survey Wald CI: 0.696 (0.493, 0.983) 0.0398 |
| ATE | Robust CI: 0.664 (0.479, 0.921) 0.0141;  Survey Wald CI: 0.664 (0.479, 0.921) 0.0141 |

Note:

Results in table: HR (95%CI) P value
IPTW: inverse probability of treatment weighting using the propensity score.

PS0: propensity score calculated by Age, HF needed, NE needed, CRP(smooth), Lymphocyte counts (smooth), SOFA(smooth), Abdominal infection.
Confounders: Age, HF needed, NE needed, CRP(smooth), Lymphocyte counts (smooth), SOFA(smooth).
PS1: propensity score calculated by confounders: Age, HF needed, NE needed, CRP(smooth), Lymphocyte counts (smooth), SOFA(smooth).

ATT: average treatment effect for treated. 
ATC: average treatment effect for control. 
ATE: average treatment effect for all.

Supplementary table-6 Summary of weights used for IPTW models

| Weight | PS | Omega-3 FA | Min. | Q1 | Median | Mean | Q3 | Max. | Sum |
| --- | --- | --- | --- | --- | --- | --- | --- | --- | --- |
| ATT | PS0 | 0 | 0.11 | 0.32 | 0.45 | 0.50 | 0.59 | 2.29 | 210.35 |
| ATT | PS0 | 1 | 1.00 | 1.00 | 1.00 | 1.00 | 1.00 | 1.00 | 211.00 |
| ATC | PS0 | 0 | 1.00 | 1.00 | 1.00 | 1.00 | 1.00 | 1.00 | 422.00 |
| ATC | PS0 | 1 | 0.12 | 1.31 | 1.73 | 1.95 | 2.38 | 6.30 | 411.20 |
| ATE | PS0 | 0 | 0.74 | 0.88 | 0.96 | 1.00 | 1.06 | 2.19 | 421.57 |
| ATE | PS0 | 1 | 0.37 | 0.77 | 0.91 | 0.98 | 1.13 | 2.43 | 207.40 |
| ATT | PS1 | 0 | 0.11 | 0.33 | 0.46 | 0.50 | 0.60 | 1.74 | 209.04 |
| ATT | PS1 | 1 | 1.00 | 1.00 | 1.00 | 1.00 | 1.00 | 1.00 | 211.00 |
| ATC | PS1 | 0 | 1.00 | 1.00 | 1.00 | 1.00 | 1.00 | 1.00 | 422.00 |
| ATC | PS1 | 1 | 0.12 | 1.35 | 1.76 | 1.95 | 2.29 | 6.05 | 412.47 |
| ATE | PS1 | 0 | 0.74 | 0.88 | 0.97 | 1.00 | 1.07 | 1.82 | 420.69 |
| ATE | PS1 | 1 | 0.37 | 0.78 | 0.92 | 0.98 | 1.10 | 2.35 | 207.82 |

Note: *：Omega-3 FA: Omega-3 fatty acid. IPTW: inverse probability of treatment weighting using the propensity score. PS: propensity score.

Supplementary table-7 Multivariable analysis

Outcome: DEATH 
Time: ICUSTAY

|  | coef. | se(coef.) | z | HR=exp(coef) | 1/HR | 95%CI lower | 95%CI upper | P　value |
| --- | --- | --- | --- | --- | --- | --- | --- | --- |
| factor(Omega-3 FA) ^*^ | -0.4815 | 0.1653 | -2.9128 | 0.6179 | 1.6185 | 0.4469 | 0.8543 | 0.0036 |
| Age | 0.0147 | 0.0047 | 3.0902 | 1.0148 | 0.9854 | 1.0054 | 1.0243 | 0.0020 |
| CRP | 0.0019 | 0.0008 | 2.3272 | 1.0019 | 0.9981 | 1.0003 | 1.0035 | 0.0200 |
| Lymphocyte count | -0.0879 | 0.0835 | -1.0529 | 0.9158 | 1.0919 | 0.7775 | 1.0787 | 0.2924 |
| HF needed | 0.2476 | 0.1630 | 1.5190 | 1.2810 | 0.7806 | 0.9306 | 1.7633 | 0.1288 |
| NE needed | 0.4031 | 0.1812 | 2.2243 | 1.4964 | 0.6683 | 1.0491 | 2.1345 | 0.0261 |
| Abdominal infection | 0.1857 | 0.3913 | 0.4745 | 1.2041 | 0.8305 | 0.5591 | 2.5928 | 0.6351 |

Note: *：Omega-3 FA: Omega-3 fatty acid. Coef.: Coefficient. Se: Standard error. HR: hazard ratio. Z: z score.
